# Supplementary material for: Optocapillarity-driven assembly and reconfiguration of liquid crystal polymer actuators
Source: Nat Commun. 2020 Nov 13;11:5780. doi: 10.1038/s41467-020-19522-1 (PMC7666155; doi:10.1038/s41467-020-19522-1)
Supplement: Supplementary file 1 — Supplementary Information [file 41467_2020_19522_MOESM1_ESM.pdf]

Supplementary Information

for

Optocapillarity-driven assembly and reconfiguration of liquid crystal polymer actuators

by Hu, *et al.*

Supplementary Figures

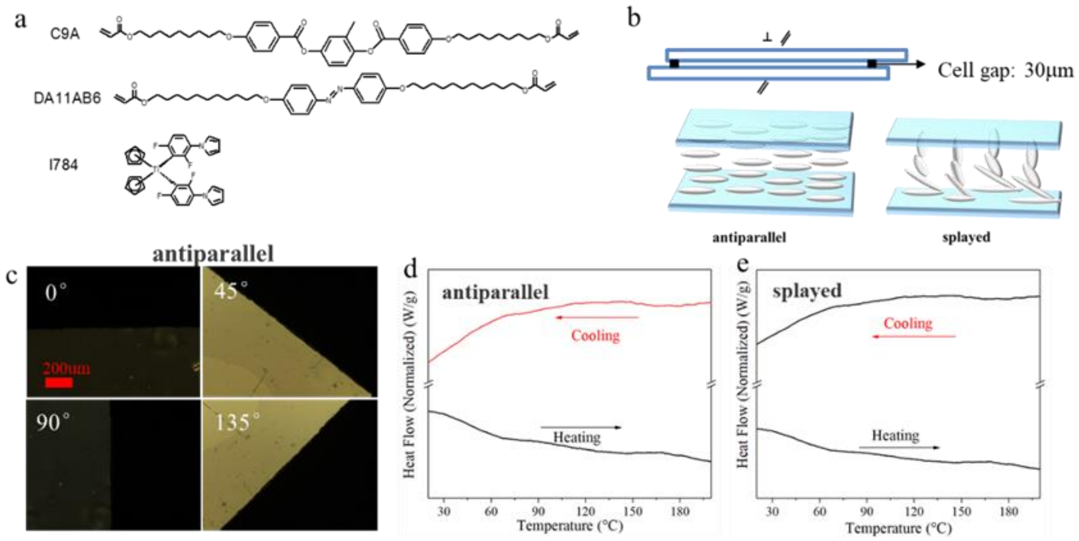

Supplementary Figure 1. Chemical structures of monomers and differential scanning calorimetry (DSC) curves of the liquid crystal network (LCN). (**a**) The Chemical structures of monomers and photoinitiator, (**b**) Schematic diagram of the orientation of liquid crystals in the cell, (**c**) Polarizing optical microscope (POM) images of the LCN actuator with antiparallel orientation, (**d**) DSC curves of antiparallel oriented LCN, (**e**) DSC curves of splay oriented LCN.

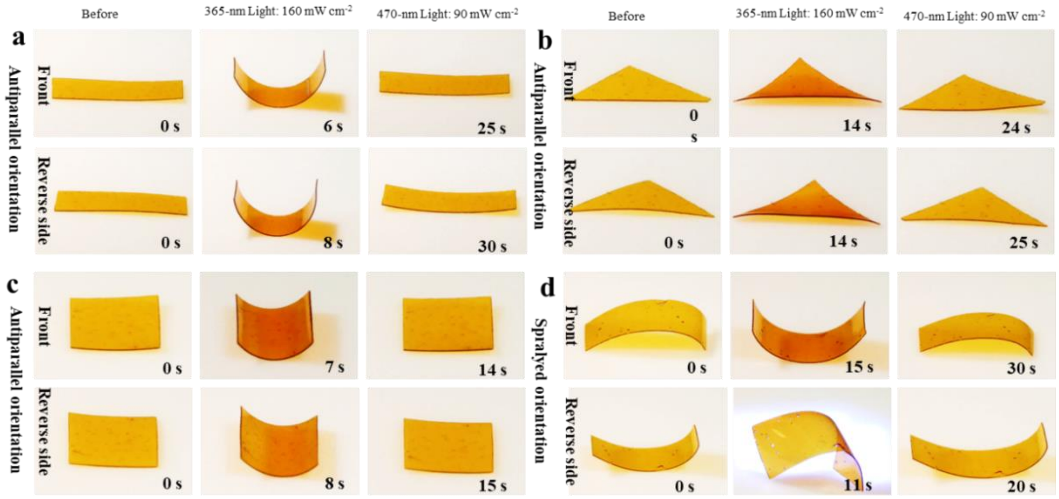

Supplementary Figure 2. Light-driven bending of azo-functionalized liquid crystal polymer actuators with different orientations and shapes. (**a-c**) Photodeformation of antiparallel oriented azo-functionalized liquid crystal polymer actuators with different shapes upon the irradiation of 365-nm and 470-nm light, respectively. (**d**) Photodeformation of the splay-oriented actuators upon the irradiation of 365-nm and 470-nm light, respectively. The intensity of 365-nm and 470-nm light is 175 mW cm^{-2} and 150 mW cm^{-2}, respectively.

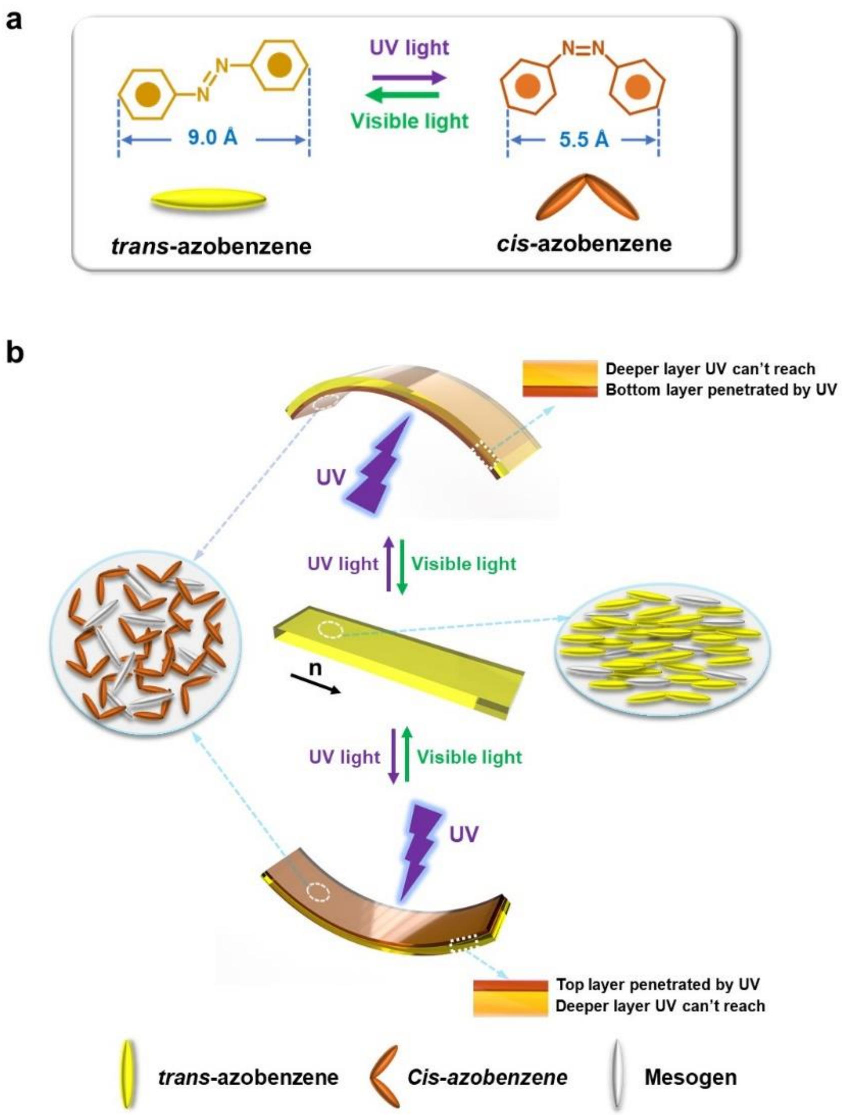

Supplementary Figure 3. **Schematics showing photodeformation mechanism of the LCN**. (**a**) Schematics showing the isomerization of azobenzene. Rod-shaped trans-azobenzene with molecular length of 9.0 Å absorbs UV light and transforms to bent cis-azobenzene with molecular length of 5.5 Å. Irradiated by visible light, cis-azobenzene backs to trans-azobenzene. (**b**) Schematics showing light-induced directional bending of the LCN with homogeneous alignment. When its bottom surface is illuminated by UV light, the LC-to-isotropic phase transition of the bottom surface causes the anisotropic contraction along the long axis of the LCN, which generate the strain mismatch between the top layer penetrated by UV and deeper layer that UV can't reach. This strain mismatch leads to downward bending of the LCN toward the incident direction of the actinic UV light. In the same way, when the top surface of LCN film is illuminated by UV light, the strain mismatch between the UV-penetrated layer and the deeper layer that UV cannot reach makes the LCN bending upwards.

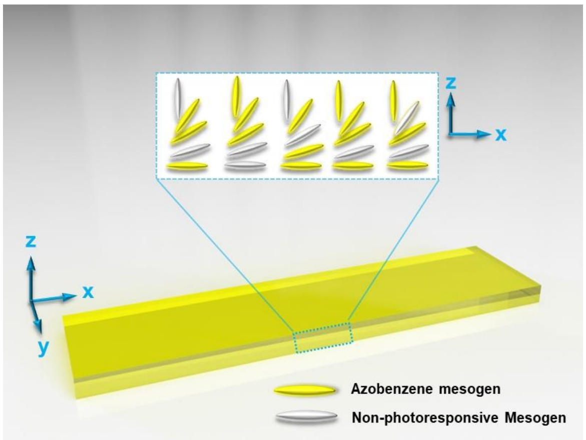

Supplementary Figure 4. Schematics showing splayed alignment in the LCN. The small dashed rectangle indicates the xz plane of the LCN. The inset enclosed in an enlarged dashed rectangle shows the splayed alignment of LC mesogens: the nematic director of LC mesogens tilts and varies in the xz plane of the LCN, the tile angle is gradually changed from 0° at the bottom to 90° at the top.

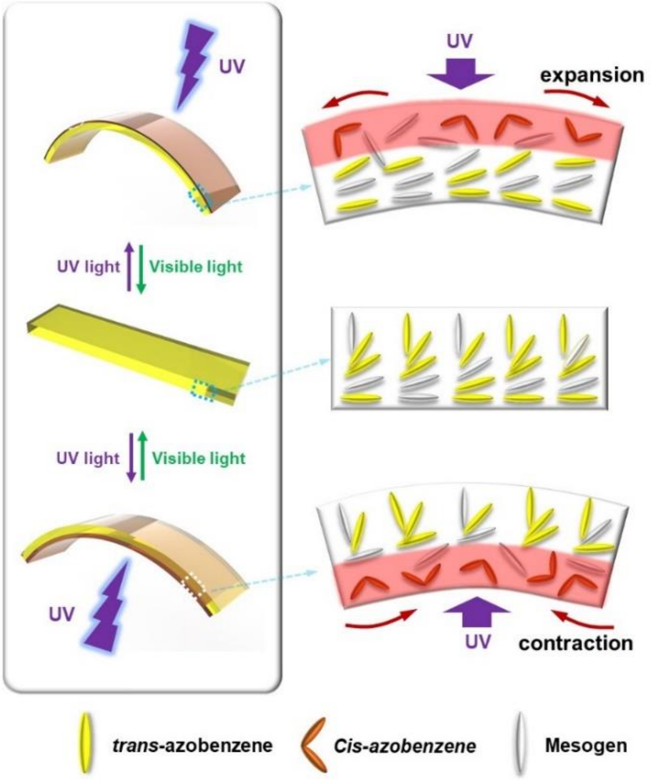

Supplementary Figure 5. Schematics showing light-driven bending of the LCN with splayed alignment. When the top surface of LCN is illuminated by UV light, the LC-to-isotropic phase transition causes the anisotropic expansion along the long axis of the LCN owing to out-plane alignment of LCs at its top surface, which generate the strain mismatch between the top layer penetrated by UV and deeper layer that can't reach by UV. This strain mismatch leads to downward bending of the LCN away the incident direction of the actinic UV light. When its bottom surface is illuminated by UV light, the LC-to-isotropic phase transition generates the anisotropic contraction along the long axis of the LCN due to in-plane alignment of LCs at the bottom surface of LCN, and the strain mismatch between the layer penetrated by UV and deeper layer leads to downward bending.

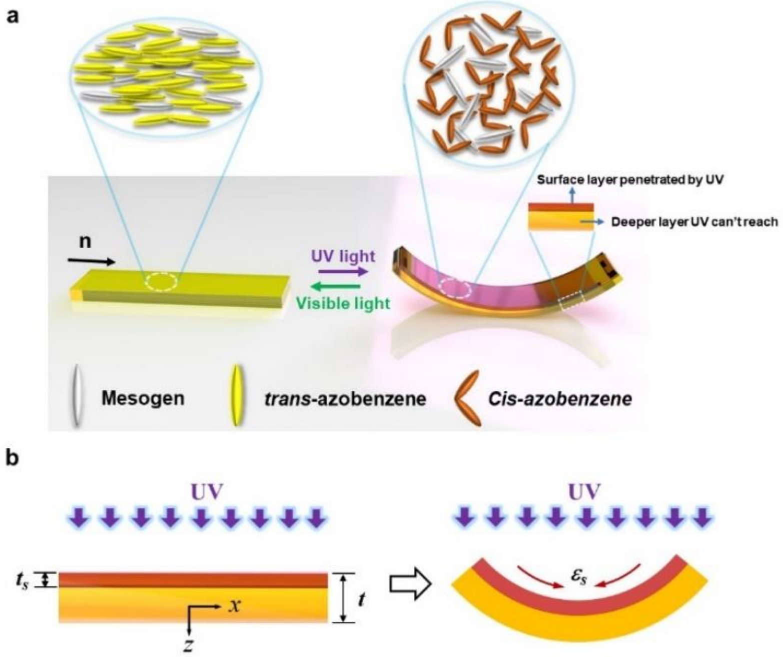

Supplementary Figure 6. Schematics showing the photodeformation model of azo-functionalized LCN. (a) light-induced reversible bending of homogeneous azo-LCN. Upon UV irradiation, LC-to-isotropic phase transition occur only in the surface region of the LCN, which result in an uneven distribution of the molecular alignment through the thickness of the sheet, and the gradient of photo-induced strains through thickness induce the bending motion toward the incident direction of the actinic UV light. (**b**) Schematics showing light-induced bending model of the LCN, which can be regarded as a bilayer: a surface layer with constant photostrain and a deeper layer without photostrain.

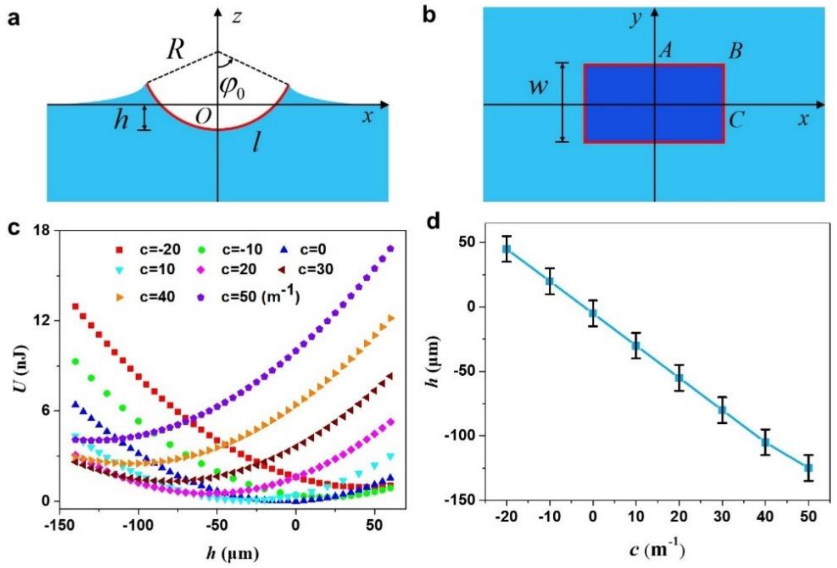

Supplementary Figure 7. Gibbs free energy in the system composed of a film and water. (**a**) Lateral view and (**b**) top view of the photodeformable liquid crystal polymer film actuator on water surface. (**c**) The Gibbs free energy variation with respect to the height of the film with different bending curvature. (**d**) The height versus the bending curvature of the film when the system reaches the steady state. The data points are the averages of the results calculated three times with different triangular meshes generated randomly, and the error bars represent the deviation from the average value.

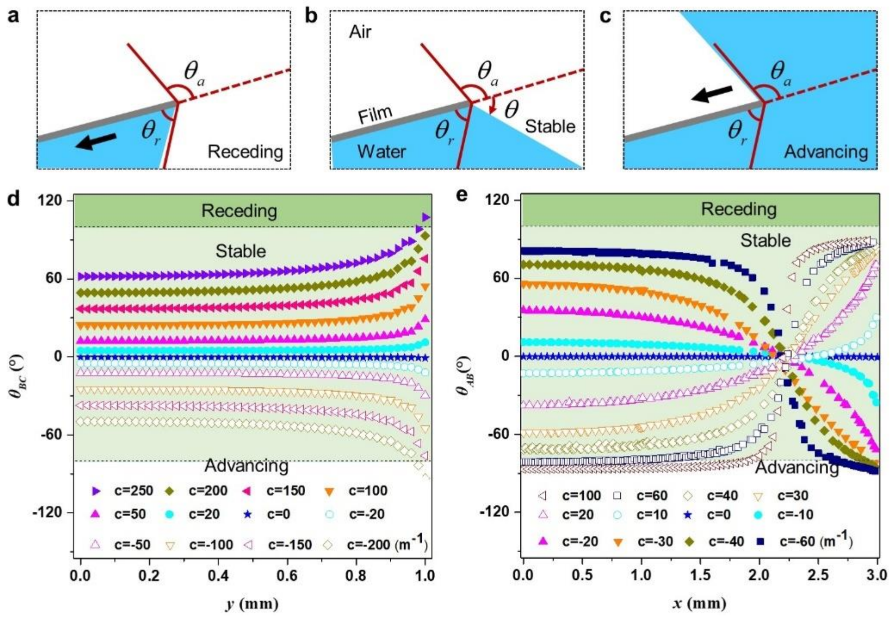

Supplementary Figure 8. Stability analysis of the three-phase line with different film curvatures. (**a**) From left to right, the three-phase line recedes, pins, and advances the film surface. (**b**, **c**) The distribution of the angle θ along the boundary BC (θ_{BC}) and AB (θ_{AB}).

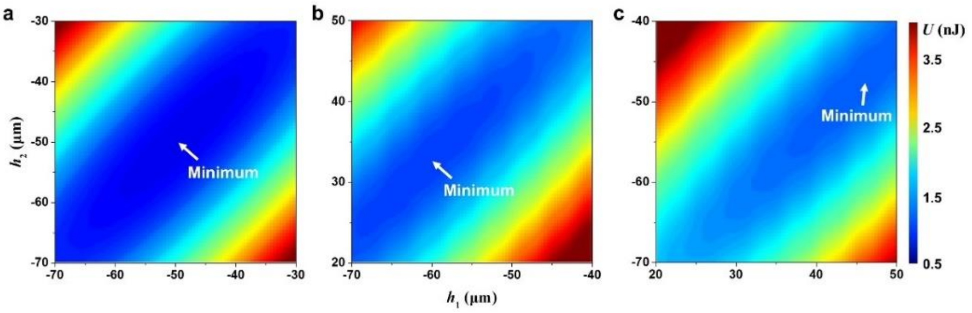

Supplementary Figure 9. Analysis of the Gibbs free energy of the two-film system. (**a-c**) The Gibbs free energy variation with the heights of the two films, corresponding to Case 1 ($c_1 = c_2 = 20$ m^{-1}), Case 5 ($c_1 = 20$ m^{-1}, $c_2 = -20$ m^{-1},), and Case 6 ($c_1 = -20$ m^{-1}, $c_2 = 20$ m^{-1}), respectively.

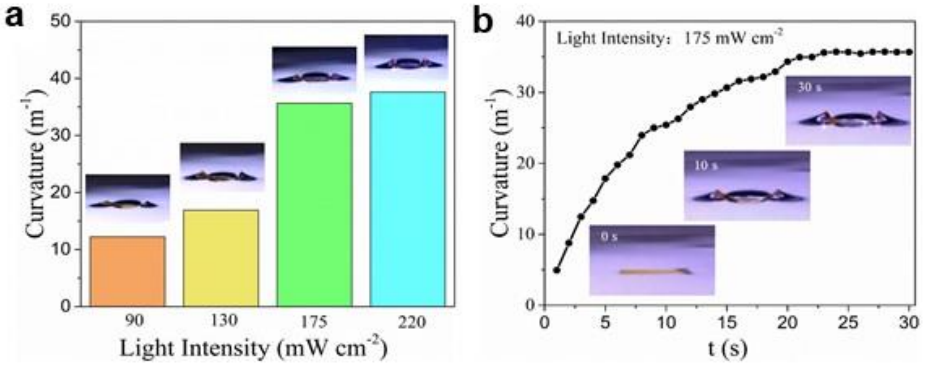

Supplementary Figure 10. Light-driven modulation of bending curvature. (**a**) Plot showing the bending curvature of the actuators when the intensity of the UV light source is varied. **(b)** Plot showing the bending curvature versus irradiation time under the same light intensity.

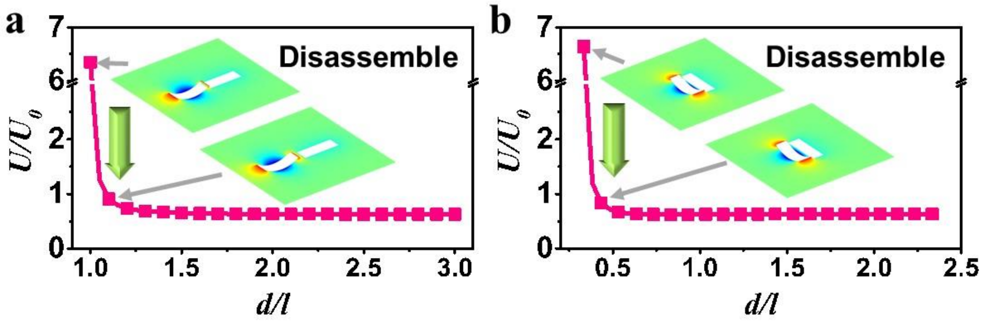

Supplementary Figure 11. **Mechanism of optocapillarity-driven disassembly.** The total Gibbs free energy of the system composed of an up-bending actuator and a flat actuator varies with the distance d. The α for the two actuators in (**a**) and (**b**) is 0° and 180°, respectively. There is a constant attraction force between two up-bending actuators when they are in end-to-end or side-by-side assembly. When one of them is exposed to the visible light and flattened, the capillary attraction will disappear, but a capillary repulsion occurs, resulting in disassembly of them. The repulsion is related to the curvature of the bending actuator and drops rapidly with the increase of their distance.

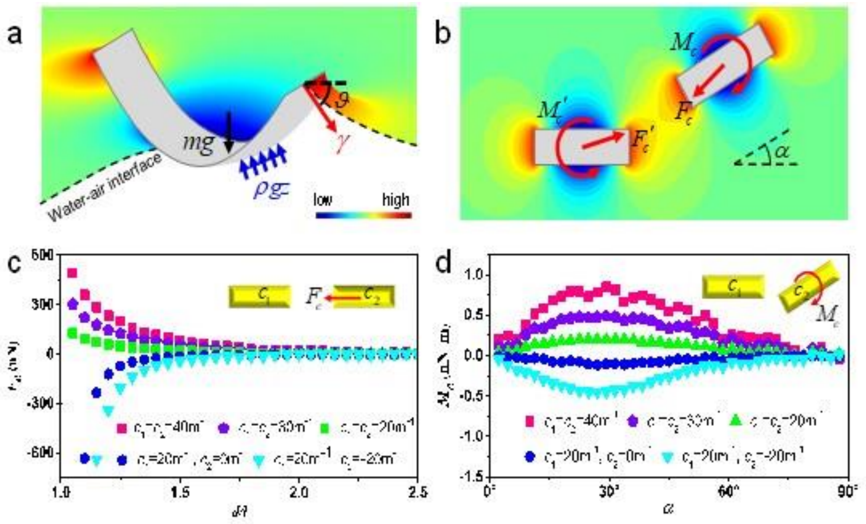

Supplementary Figure 12. Dynamic analysis of the floating actuators. (**a**) Force analysis of the single floating actuator. (**b**) Capillary interaction between two floating actuators. (**c**) Numerical data for capillary force F_c on the actuator (with curvature c_2) vs relative distance d/l from another one (c_1). Here the long axes of the two actuators are in a line, as shown in the inset. (**d**) Numerical data for capillary moment M_c on the actuator (c_2) vs the orientation angle α with another one (c_1). Here the center of the c_2 actuator is on the long axis of the c_1 actuator and the center-to-center distance is $d = 1.2l$, as shown in the inset

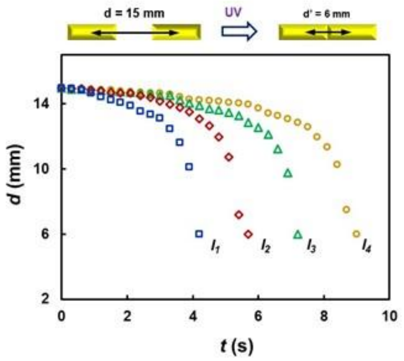

Supplementary Figure 13. Plots showing center-to-center distance between two actuators versus irradiation time when the intensity of UV light source is different. I_1 = 170 mW cm^{-2} (blue squares), I_2 = 155 mW cm^{-2} (red rhombs), I_3 = 120 mW cm^{-2} (green triangles), and I_4 = 90 mW cm^{-2} (orange circles). Schematics above showing experiment setup. The initial center-to-center distance of two actuators is fixed at 15 mm, the distance decreases to 6 mm when they finish the end-to-end assembly upon light irradiation. The size of the actuators is 6 mm × 2 mm × 0.03 mm.

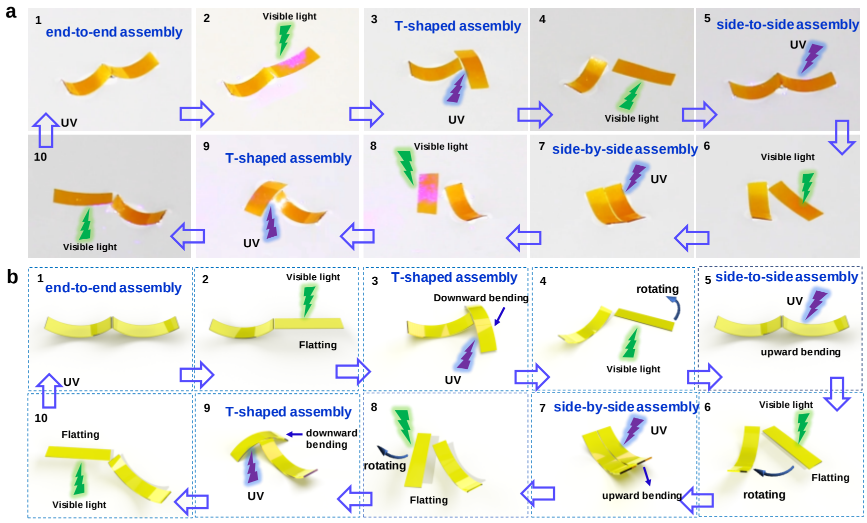

Supplementary Figure 14. Experimental photographs (a) and corresponding Schematics (b) showing optocapillarity-driven reconfiguration of two actuators. To achieve the reconfiguration from end-to-end assembly (a1) to T-shaped assembly (a3), one actuator was illuminated with visible light to make it flat (a2, b2), and then its bottom surface was irradiated with UV light (a3, b3), and the actuator bent downwards, then the two actuators aggregated into T-shaped assembly. For the reversible reconfiguration, we can firstly illuminate downward bending actuator with visible light to recover to flat (a4, b4), and then the flattened actuator rotated (a4, b4). When the orientation angle was less than 60°, the flattened actuator was illuminated UV light to bend upwards, and then the two actuators reaggregated back into end-to-end assembly (a5). To achieve the reconfiguration from end-to-end assembly to side by side assembly, firstly we can illuminate one actuator with visible light to make it flat, and the flattened actuator rotated (a6, b6). When the orientation angle was large than 120°, the flattened actuator was illuminated with UV light to let it bend upwards, and then the two actuators formed side by side assembly (a7, b7). To gain the reconfiguration from side by side assembly to T-shaped assembly, one actuator was irradiated with visible light to become flat (a8, b8). The flattened actuator was irradiated with UV light at the bottom surface to make it bend downwards. Then the two actuators aggregated into T-shaped assembly (a9, b9). Using the same methods described above, the T-shaped assembly can be reconfigured again into end-to-end assembly (a9-a10, b9-b10).

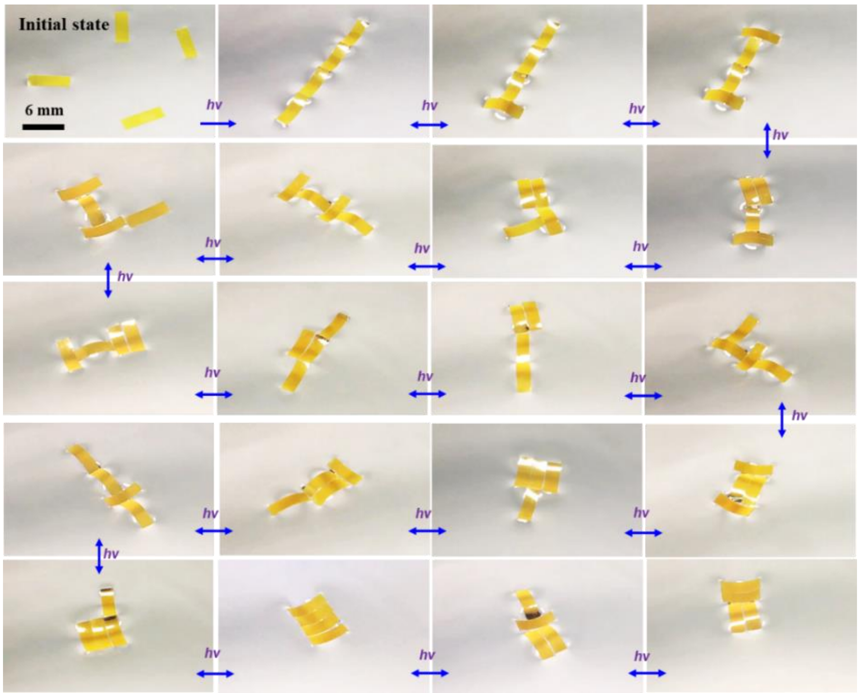

Supplementary Figure 15. Optocapillarity-driven programmable and reconfigurable assembly. Photographs showing light-induced assembly of four rectangular actuators into 19 ordered structures. The first image exhibits the initial location of the actuators before light irradiation. Upon light irradiation, the actuators assemble into regular structures. The transformation between any two structures is achieved by alternative localized irradiation of 450-nm and 360-nm laser as well as modulation of the incident direction of the laser. The intensity of the ultraviolet laser and visible laser is 60 mW cm^{-2} and 45 mW cm^{-2}, respectively. The size of rectangular actuators is 6 mm × 2 mm × 0.03 mm.

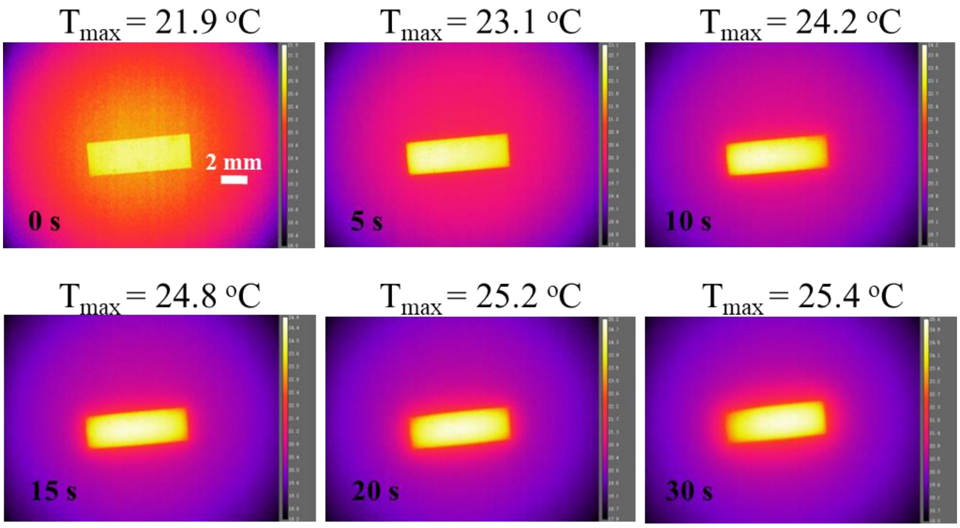

Supplementary Figure 16. Temperature of actuator at the air-water interface before and upon illumination of 365-nm light. The intensity of 365-nm light is 175 mW cm^{-2}. The size of actuator is 6 mm × 2 mm × 0.03 mm.

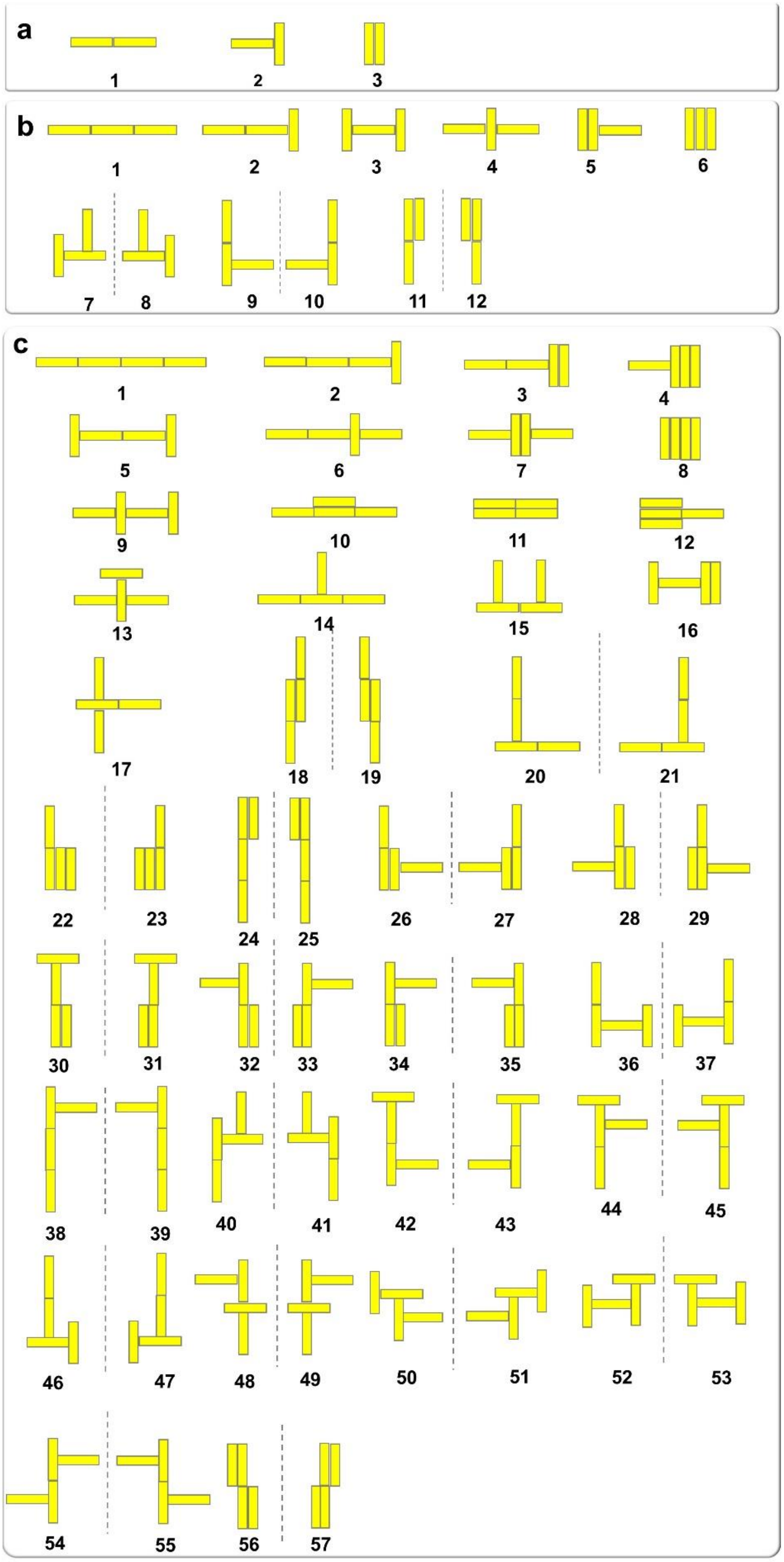

Supplementary Figure 17. Schematics showing light-driven reconfigured structures by 2 actuators (a), 3 actuators (b), and 4 actuators (c), respectively. Interestingly, 6 structures formed by 2 actuators and 40 structures formed by 3 actuators show chirality. The couples of the actuators at the left and right sides of the dotted lines are enantiomers that constitute mirror images that cannot be superimposed on each other.

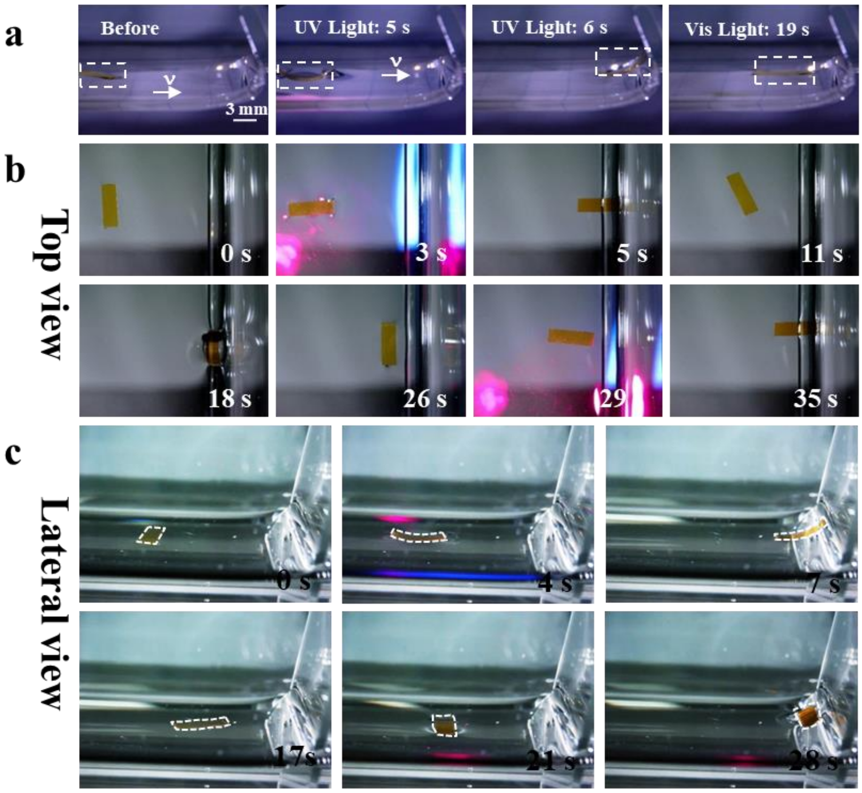

Supplementary Figure 18. Optocapillarity-driven meniscus-climbing. (**a**) Time-lapse images showing light-driven reversible climbing of meniscus (Supplementary Movie 6). Initially, a flat actuator is placed at the foot of the meniscus. When irradiated with UV light (175 mW cm^{-2}), the actuator bent downward and rotated to align itself perpendicular to the meniscus, and then accelerated and ascended the meniscus. And upon irradiation of 470-nm light (150 mW cm^{-2}), the actuator unbends and slide down the meniscus. The top view (**b**) and lateral view (**c**) of meniscus climbing and rotating. Size of the actuator is 6 mm × 2 mm × 0.03 mm.

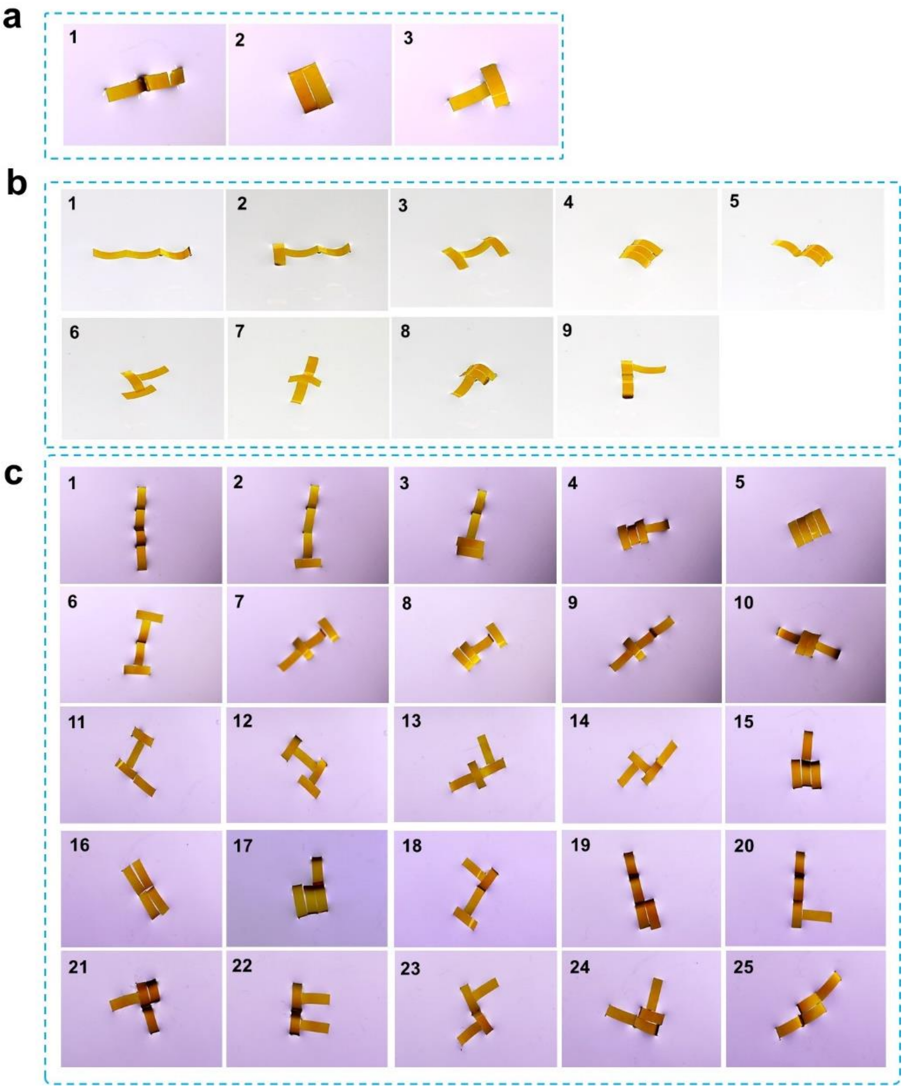

Supplementary Figure 19. Experimental photographs showing light-driven reconfigured structures by 2 actuators (a), 3 actuators (b), and 4 actuators (c), respectively. Only some representative structures for 4 actuators are shown in **c** because there are many different forms.

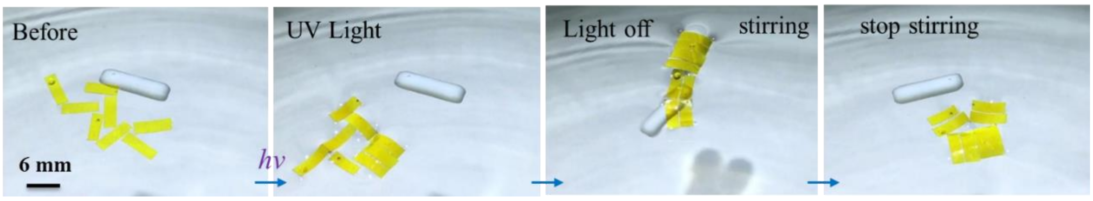

Supplementary Figure 20. Time-sequence photographs showing selective aggregation controlled by light. There are eight actuators randomly dispersed at the interface. Illumination of UV light at any four actuators leads them to aggregate together to form a strong-binding array that enable resist the shearing force induced by the stirring while the rest four actuators illumination of UV light from down are combine in different arrangements from each other by the stirring. The size of individual actuator is 6 mm × 2 mm × 0.03 mm. The intensity of 365-nm and 470-nm light is 175 mW cm^{-2} and 150 mW cm^{-2}, respectively. Rotating speed of the magnetic agitator is 200 rpm.

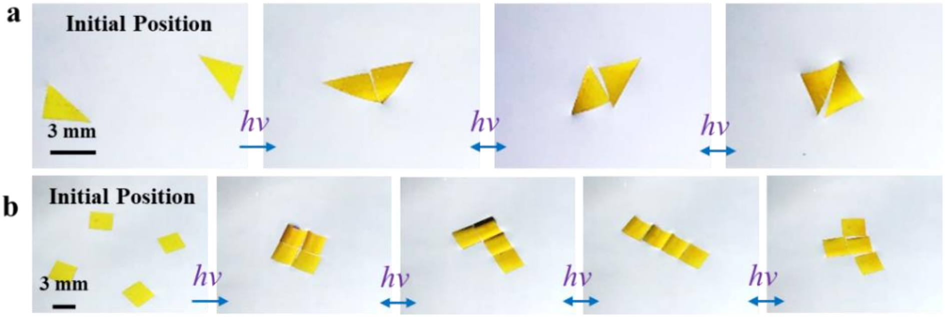

Supplementary Figure 21. Versatility of Optocapillarity-driven assembly. a. Photographs showing two triangular actuators assembled into isosceles, triangular, rhombic, and square structures. **b.** Photographs showing four square actuators assembled aggregated into L", "I", and "T"-shaped structures. The first image in each row of images exhibits the initial location of the actuators before light irradiation. Upon light irradiation, the actuators assemble into regular patterns. The transformation of the structures was achieved by alternative localized irradiation of 450-nm and 360-nm laser with modulation of the incident direction. The intensity of the ultraviolet laser and visible laser is 60 mW cm^{-2} and 45 mW cm^{-2}, respectively. The size of isosceles right triangular actuators and square actuators is 3 mm × 0.03 mm and 3 mm × 3 mm × 0.03 mm, respectively.

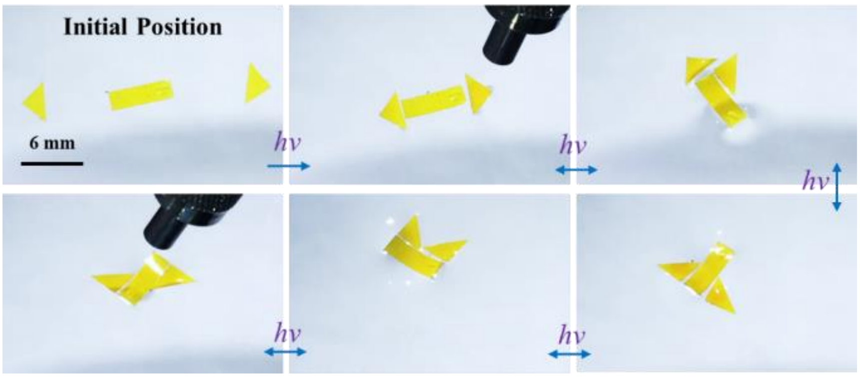

Supplementary Figure 22. Photocontrol of two triangular actuators and a rectangular actuator to assemble into structures like indicatory signs. The first image exhibits the initial location of the actuators before light irradiation. Upon light irradiation, the actuators assemble into regular patterns. The transformation of the structures was achieved by alternative localized irradiation of 450-nm and 360-nm laser with modulation of the incident direction. The intensity of the ultraviolet laser and visible laser is 60 mW cm^{-2} and 45 mW cm^{-2}, respectively. The size of isosceles right triangular actuators, rectangular actuators is 3 mm × 0.03 mm, 6 mm × 2 mm × 0.03 mm, respectively.

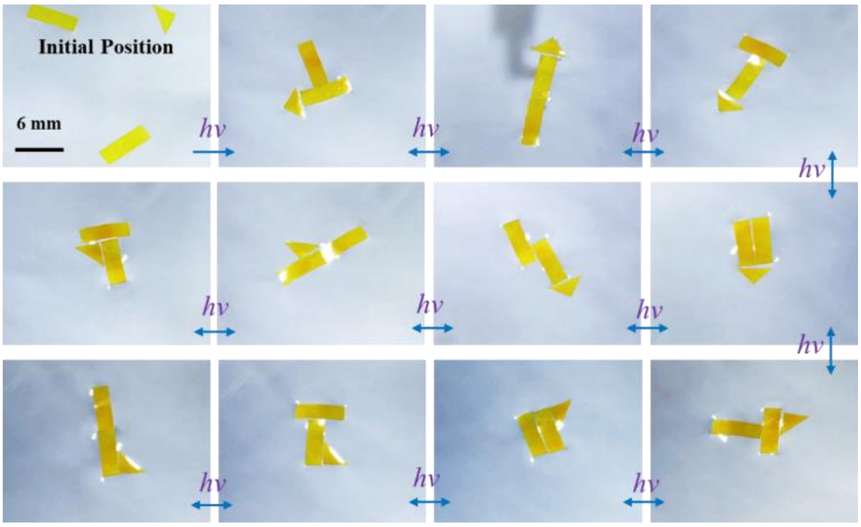

Supplementary Figure 23. Photocontrol of a triangular actuator and two rectangular actuators to assemble into structures like indicatory signs. The first image exhibits the initial location of the actuators before light irradiation. Upon light irradiation, the actuators assemble into regular patterns. The transformation of the structures was achieved by alternative localized irradiation of 450-nm and 360-nm laser with modulation of the incident direction. The intensity of the ultraviolet laser and visible laser is 60 mW cm^{-2} and 45 mW cm^{-2}, respectively. The size of isosceles right triangular actuators, rectangular actuators is 3 mm × 0.03 mm, 6 mm × 2 mm × 0.03 mm, respectively.

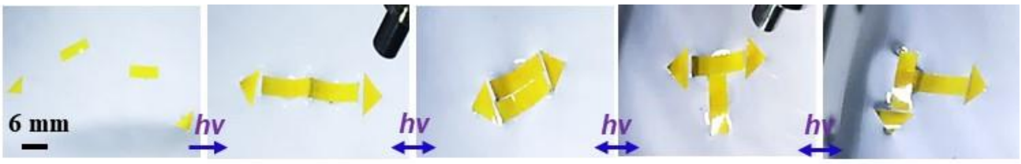

Supplementary Figure 24. Photocontrol of two triangular actuators and two rectangular actuators to assemble into structures like indicatory signs. The transformation of the structures was achieved by alternative localized irradiation of 450-nm and 360-nm laser with modulation of the incident direction. The intensity of the ultraviolet laser and visible laser is 60 mW cm^{-2} and 45 mW cm^{-2}, respectively. The size of isosceles right triangular actuators, rectangular actuators is 3 mm × 0.03 mm, 6 mm × 2 mm × 0.03 mm, respectively.

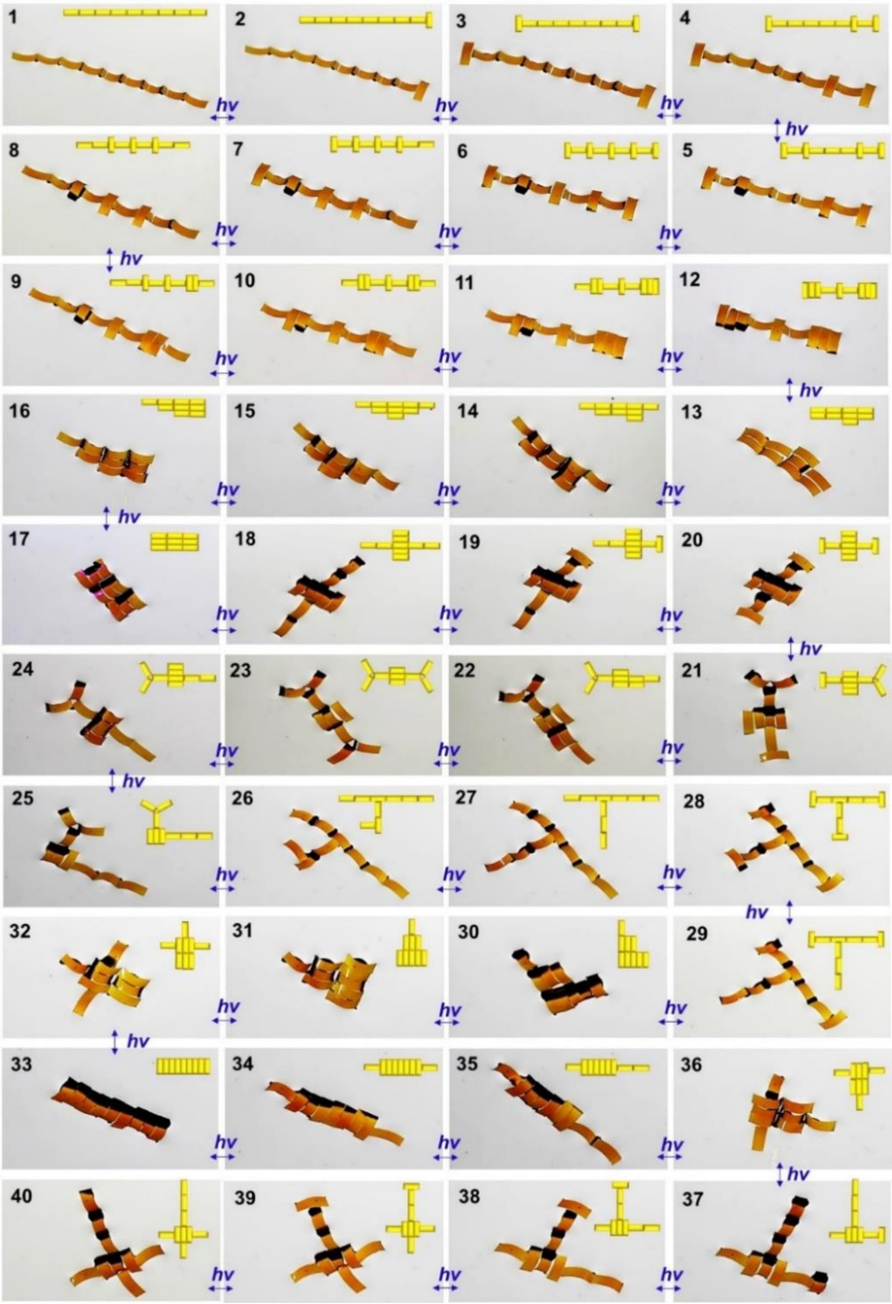

Supplementary Figure 25. Optocapillarity-driven programmable reconfiguration. Photographs showing light-induced assembly and reconfiguration of nine rectangular actuators into diverse structures. Upon light irradiation, the actuators assemble into regular structures. The transformation between any two structures is achieved by alternative localized irradiation of 450-nm and 360-nm laser as well as modulation of the incident direction of the laser (Supplementary Movie 8). The inset at the top right corner of each photograph schematically indicates the assembled structure. The intensity of the ultraviolet laser and visible laser is 60 mW cm^{-2} and 45 mW cm^{-2}, respectively. The size of rectangular actuators is 6 mm × 2 mm × 0.03 mm.

Supplementary Tables

Supplementary Table 1. List of parameters used in the numerical calculation

Symbol	Parameter	Value	Unit
l	Length of the film	6e-3	m
w	Width of the film	2e-3	m
	Thickness of the film	3e-5	m
ρ	Density of water	998	kg m^{-3}
γ	Surface tension of water	7.28e-2	N m^{-1}
	Density of the film	1.2ρ	
g	Gravity acceleration	9.81	kg m s^{-2}

Supplementary Table 2. Dynamic and static contact angles of the liquid crystal polymer actuators

**contact angles**	**Static contact angles**			**Dynamic contact angles** Before			UV 60s		
	Before (°)	UV 30s (°)	Vis 30s (°)	θ_a (°)	θ_r (°)	$\Delta\theta$ (°)	θ_a (°)	θ_r (°)	$\Delta\theta$ (°)
Antiparallel oriented LCP	72.01±1.4	70.98±1.6	72.32±1.5	75.79±1.4	65.13±2.3	10.66±2.4	72.53±1.4	64.67±1.5	7.86±1.9
Splayed oriented LCP	79.84±0.5	78.58±0.5	79.76±0.6	79.17±1.4	66.63±1.2	12.54±3.3	78.75±1.3	67.72±0.5	11.03±1.8

θ_a, θ_r, $\Delta\theta$ represent advancing angle, receding angle and the difference between advancing angle and receding angle on flat LCP films. Data are from five individual measurements of each variable. Errors, s.d. The intensity of 365-nm and 470-nm light is 175 mW cm^{-2} and 150 mW cm^{-2}.

Supplementary Note 1

Azobenzene can undergo a reversible photochemical reaction between two isomers (Supplementary Fig. 3a). Rod-shaped trans-azobenzene with molecular length of 9.0 Å absorbs UV light and transforms to bent cis-azobenzene with molecular length of 5.5 Å. Irradiated by visible light, cis-azobenzene can back to *trans*-azobenzene. Rod-shaped *trans*-azobenzenes stabilize the ordered phase structure of the LC, while the bent *cis*-azobenzenes tend to destabilize the phase structure [1]. When azobenzenes are incorporated into liquid crystal network (LCN) as photoresponsive mesogens, the photochemical reaction of azobenzene can be used to isothermally trigger reversible LC-to-isotropic phase transition of the LCN [2]. Azobenzene possesses a high extinction coefficient around 360 nm (about 2.0×10^4 L mol^{-1} cm^{-1}). High concentration of azobenzene in the LCN film causes extensive absorption of the incident photons at the surface region (99% incident photons are absorbed by the surface within a thickness of < 1 μm), which means that LC-to-isotropic phase transition occurs only in the surface region of the LCN upon UV irradiation [3].

In the homogeneous LCN, LC mesogens are aligned parallel to the long axis of the LCN (Supplementary Fig. 3b). When the top surface of LCN is illuminated by UV light, the LC-to-isotropic phase transition of the top surface causes the anisotropic contraction along the long axis of the LCN, which generates the strain mismatch between the top layer penetrated by UV and deeper layer that UV can't reach. This mismatch of photo-induced strain leads to upward bending of LCN toward the incident direction of the actinic UV light. In the same way, when its bottom surface is illuminated by UV light, the mismatch between the UV-penetrated layer and the deeper layer that UV can't reach makes the LCN bending downwards.

Splayed alignment describes a director profile of LC mesogens aligned in a tilted orientation (Supplementary Fig. 4), in which the nematic director of LC tilts and varies in the *xz* plane, the tile angle is gradually changed from 0° at the bottom to 90° at the top. As shown in Supplementary Fig. 5, when the top surface of LCN film with splayed alignment is illuminated by UV light, the LC-to-isotropic phase transition of the top surface causes the anisotropic expansion along the long axis of the LCN owing to out-plane alignment of LCs at the top surface of LCN, which generates the strain mismatch between the top layer penetrated by UV and deeper layer that UV can't reach. This mismatch of photo-induced strain leads to downward bending of LCN away the incident direction

of the actinic UV light. While its bottom surface is illuminated by UV light, the LC-to-isotropic phase transition of the bottom surface generates the anisotropic contraction along the long axis of the LCN owing to in-plane alignment of LCs at the bottom surface, then the mismatch of photo-induced strain leads to downward bending.

Supplementary Note 2

The precision of the assembly is controlled by the bending of actuators. When the bending direction of two actuators is opposite, one bends upwards and the other bends downwards, they always adopt T-shaped assembly. When two actuators have the same bending direction, both bend upwards or downwards. The resulted state of assembly will be affected by initial distance between them. There is a critical distance that determines assembly states. As shown in Fig. 1d in the main paper, under the premise of radiating light with the same intensity (110 mW cm^{-2}) and placing two actuators in parallel, when the initial distance between two actuators is greater than the critical distance approx. 11.2 mm, two actuators form end to end assembly, while the distance is less than the critical distance and they would like to aggregate into side-by-side.

Supplementary Note 3

The bending of azo-functionalized liquid crystal polymer actuators (LCN films) are caused by the light-induced uneven shrinkage deformation along the thickness direction (Supplementary Fig. 6). When the film is illuminated by UV light from the top, due to the strong capacity of photo absorption of the material containing azobenzene groups, the light intensity would decay exponentially along the thickness direction, that is [4]

$$I = I_0 e^{-[z+(t/2)]/d} \tag{1}$$

Here I_0 is the light intensity at the top surface, z is the distance from the top surface, t is the thickness of the film, and d is the attenuation coefficient. Upon UV irradiation, LC-to-isotropic phase transition occur to generate negative photostrain within the film. The photostrain is determined by the light intensity I and the light duration T [4-6]. Assume the photostrain is proportional to the light intensity, we have

$$\varepsilon^p = \varepsilon_0^p (I_0, T) e^{-[z+(t/2)]/d} \tag{2}$$

Here $\varepsilon_0^p(I_0,T)$ is the in-plane photostrain at the top surface. According to ref. 4, the deformation induced by the above photostrain gradient can be approximately calculated as a bilayer, which is composed of a surface layer with constant photostrain and a deeper layer without photostrain, as shown in Supplementary Fig. 6b. The thickness and the value of photostrain of the surface layer can be respectively expressed as

$$t_s = 2d - \frac{t}{e^{t/d} - 1} \tag{3}$$

$$\varepsilon_s = \varepsilon_0^p(I_0,T)\frac{(e^{t/d}-1)^2}{2e^{t/d}\left[(e^{t/d}-1)d - t\right]} \tag{4}$$

In the absence of external forces, the film will form a cylindrical shape with curvature [4].

$$c = \frac{6\varepsilon_s t_s(t_s - t)}{\pi t^3} = \frac{3\left(2de^{t/d} - 2d - t\right)\left(2de^{t/d} - 2d - te^{t/d}\right)}{\pi t^3 e^{t/d}\left(de^{t/d} - d - t\right)}\varepsilon_0^p(I_0,T) \tag{5}$$

Therefore, the curvature of the film will depend on the light intensity and light duration.

Supplementary Note 4

In our experiments, the deformation of the actuator is mainly regulated by the light field. To facilitate the understanding, the actuators can be regarded as rigid bodies during their assembly process. Here we assume that the shape of the film keeps as a segment of circle and the length remains unchanged.

For the curving film, let c denote the curvature, l the arc length, and w the width. Then the radius and arc angle can be calculated by $R = c^{-1}$ and $2\varphi_0 = lc$, separately. As shown in Supplementary Fig. 7(a-b), the z axis goes up vertically, the x and y axes follow the initial directions of length and width of the film, respectively. The height of the horizontal water surface at infinity is set to be $z = 0$. In our experiments, the lower surface of films was keeping in contact with water surface until it was submerged. This means that the water-solid interface energy is a constant, without contribution to the free energy of the system composed of the solid and liquid. Hence, the Gibbs free energy of the system can be expressed as the sum of air-water interface energy, gravitational potential energy of water, and gravitational potential energy of the film, that is

$$U = \gamma \iint_{WA_\perp}\left(\sqrt{1 + z_x^2 + z_y^2} - 1\right)\mathrm{d}x\mathrm{d}y + \iint_{WS_\perp + WA_\perp}\frac{\rho g}{2}z^2\mathrm{d}x\mathrm{d}y + mgh \tag{6}$$

where $z(x,y)$ describes the water surface morphology, z_x and z_y are the partial derivatives with respect to x and y, respectively. γ and ρ are the surface tension coefficient and density of water, m is the mass of the film, g is the gravitational acceleration, and h is the height of the film bottom from the horizontal plane. $WA_{\perp}$ and $WS_{\perp}$ respectively represent the projection on the horizontal plane of air-water interface and water-solid interface.

According to force analysis of the liquid near the air-water interface, the hydrostatic pressure, ρgz, is to balance the pressure difference between the inside and outside the interface, Δp. According to Laplace equation, $\Delta p = 2\gamma H$, where H is the local mean curvature of air-water surface, we can obtain

$$\frac{(1+z_x^2)z_{yy} - 2z_x z_y z_{xy} + (1+z_y^2)z_{xx}}{(1+z_x^2+z_y^2)^{3/2}} = \frac{\rho g}{\gamma} z \tag{7}$$

Furthermore, assuming that the air-water interface satisfies small slope approximation, the quadratic and higher-order terms in the above equation can be ignored. Then Supplementary Equation 7 can be simplified as

$$z_{xx} + z_{yy} - \frac{\rho g}{\gamma} z = 0 \tag{8}$$

For two-dimensional cases, i.e., without consideration of the liquid surface deformation along y axis, Supplementary Equation 8 can be further reduced to $z_{xx} = \rho g z \gamma^{-1}$. And the equation has a theoretical solution in the form of $z = -z_0 e^{-x\sqrt{\rho g/\gamma}}$, which is consistent with the results of previous works [7, 8].

For three-dimensional cases, as shown in Supplementary Fig. 7b, there are geometric relationships on the boundary BC and AB,

$$z = R(1-\cos\varphi_0) + h \quad \left(0 \le y \le \frac{w}{2}, x = R\sin\varphi_0\right) \tag{9}$$

$$z = R + h - \sqrt{R^2 - x^2} \quad \left(0 \le x \le R\sin\varphi_0, y = \frac{w}{2}\right) \tag{10}$$

Here $h < 0$ represents that the bottom of the film is under the horizontal plane.

According to the principle of minimum potential energy, when the total free energy is minimized, the system reaches an equilibrium state. Therefore, a finite element method using the software of

matlab was applied to solve the Supplementary Equation 8-10 numerically, with optimization of Supplementary Equation 1 to make the Gibbs free energy reach minimum. For the systems with films of different bending curvature, Supplementary Fig. 7c shows the curves of the system free energy U, versus the height of the film bottom h. The local minimum of each energy curve is unique, which means the system should be monostable while the film curvature is fixed. When the system reaches stability, the height of the film bottom is denoted as the stable height. Supplementary Fig. 7d shows the relation of stable height versus the film curvature in detail. Based on this, the stable morphology of the bending actuator and air-water interface is obtained. Results of some typical configurations are shown in Fig. 3d. All value of parameters in the calculation are given in Supplementary Table 1.

The critical condition for the system to keep stable is discussed below. There are three forces acting on the film: the gravity, the hydrostatic pressure on the lower surface of the film, and the surface tension on the three-phase line (i.e. the shared boundary of water-solid and air-water interface). When the film curvature is adjusted, the summation of hydrostatic pressure changes accordingly, as well as the surface tension. Direction of surface tension are dependent on the contact angle of water on the solid surface. As shown in Supplementary Fig.8(a-c), θ is the angle between the air-water and water-solid interface, with a positive value in clockwise direction. When the contact angle on the lower solid surface, $(\pi-\theta)$, is less than the receding contact angle, θ_r, the three-phase line will move backward and part of the lower film surface will emerge from the water. In this case, the mechanics model above maybe inapplicable since the water-solid interface energy has decreased. On the other hand, when the contact angle on the upper solid surface, $-\theta$, exceeds the advancing contact angle, θ_a, the three-phase line will move forward and part of the upper film surface will be submerged. In this case, the equilibrium state of system is possible to be broken. When $-\theta_a \le \theta \le (\pi-\theta_r)$, the three-phase contact line will stay where it is and the system should be stable. In our experiments, the receding and advancing contact angle were tested and $\theta_a \approx \theta_r \approx 80^\circ$, where contact angle hysteresis effect could be neglected. Hence, to keep the three-phase line pinning the boundaries of the film, the contact angle needs to satisfy the condition, $-80^\circ \le \theta \le 100^\circ$.

Based on the results calculated from the above mechanics model, we obtain the height gradient field of air-water surface. Then according to the geometry relationships, the varied angle θ along

the boundary BC and AB (see Supplementary Fig. 7b) is calculated numerically as shown in Supplementary Fig. 8d and 8e, respectively. From the plots, we could get the following information. On the boundary BC, the three-phase line will move when the curvature of the film is out of range, (−150 m^{-1}, 200 m^{-1}). And on the boundary AB, the three-phase line will flood the tail end of film (point B) when $c > 60\ \mathrm{m}^{-1}$, but the middle of film (point A) when $c < -30\ \mathrm{m}^{-1}$. In conclusion, the film curvature should satisfy an approximate range of $-30\ \mathrm{m}^{-1} \le c \le 60\ \mathrm{m}^{-1}$ to keep it from sinking. In addition, based on our stability analysis, the upper and lower film surface with differently designed wetting abilities, e.g., a hydrophobic top and a hydrophilic bottom, will be beneficial to enhance the stability of system, which is of guiding significance to bionic design of artificial surfaces.

Supplementary Note 5

The above theoretical model can be extended to multi-film system and used to explain the phenomenon of spontaneous assembly of bending films. For a system of water poor and many films, the total Gibbs free energy can be calculated by

$$U = \gamma \iint_{WA_{\perp}} \left(\sqrt{1+z_x^2+z_y^2}-1\right)\mathrm{d}x\mathrm{d}y + \iint_{WS_{\perp}+WA_{\perp}} \frac{\rho g}{2} z^2 \mathrm{d}x\mathrm{d}y + \sum_i mgh_i \qquad (11)$$

where the last item is the sum of gravitational potential energy of all films, h_i represents the height of i-th film bottom. As mentioned before, the height fluctuation of water surface deformed by one bending film is very small (about 50 μm when $c = 20\ \mathrm{m}^{-1}$), which is different from the situation of meniscus self-climbing toward a wall (about 2 mm) [8, 9]. When another film gets close to this, the upwarp discrepancy between the nose and tail ends of two films is small and can be ignored. Hence, we assume that the noses and tails of the films are at the same height during assembly in the following numerical calculation.

When the shape of films is fixed, it was observed experimentally that there are six typical stable two-film assembly configurations on a sufficiently large pool of water, as shown in Fig 2f. To simulate these cases, the Gibbs free energy of their systems are calculated by the finite element method described above. In Supplementary Fig. 9(a-c), we show the energy contours of Cases 1, 5, and 6 versus the respective heights of the two films. The energy contours indicate that the assembled two-actuator system prefers to rise or fall synchronously due to the inappreciable Gibbs free energy

variation via that movement. According to the minimum free energy principle, the stable heights of the two films are obtained.

Supplementary Discussion

Force analysis have been performed to explain the optocapillary-driven assembly. As shown in Supplementary Fig. 12a, the forces acting on a single floating actuator can be divided into three parts, which are the gravity *mg*, the hydrostatic pressure ρgz acting on the lower surface of the actuator, and the surface tension γ acting on the three-phase line (TPL), i.e. the boundary of the actuator. Then, the resultant force on the actuator along the vertical direction and the horizontal direction are respectively

$$\vec{F} = m\vec{g} + \int_{TPL} \vec{\gamma} dl + \iint_{WS} \rho gz\vec{n} ds \tag{12}$$

$$\vec{M} = \int_{TPL} \overrightarrow{L_p} \times \vec{\gamma} dl + \iint_{WS} \rho gz\overrightarrow{L_p} \times \vec{n} ds \tag{13}$$

Here $\vec{n}$ is the local normal vector of the actuator surface, $\overrightarrow{L_p}$ is the distance vector from the center of gravity to any point on the TPL or actuator surface. Once the actuator is in equilibrium, the adjacent air-water interface has symmetric geometry (Fig. 3a in the Manuscript). When another actuator approaches, as shown in Supplementary Fig. 12b, the morphology of air-water interface changes and the symmetry is broken. Therefore, the direction of surface tension acting on the three-phase line varies accordingly, resulting in capillary force F_c and moment M_c. Noted that the contribution of hydrostatic pressure to the horizontal direction and water resistance could be negligible [7, 8], hence the horizontal motion of the actuator will be dominated by F_c and M_c. The force and moment can be calculated as the partial derivative of energy (Fig. 2b-2e in the Manuscript) with respect to the displacement and the orientation angle (the angle of long axis of the two actuators), respectively. As shown in Supplementary Fig. 12c, during the end-to-end assembly, the capillary force between two floating actuators is inversely proportional to their distance and possesses a nonlinear characteristic. For two actuators with the same curvature ($c_1 = c_2$), the greater the bending curvature, the stronger the capillary attraction force between them. However, when one of them is treated with visible light and becomes flat ($c_1 > 0$, $c_2 = 0$), the capillary force is less than zero, which gives rise to a repulsion between them, resulting in the disassembly of the end-to-end state. Furthermore, when this actuator bends in the opposite direction, the capillary repulsion gets stronger

to speed up the disassembly process. Supplementary Fig. 12d shows the capillary moment curve of one actuator with respect to the orientation angle when it is near to another one. It illustrates that the two actuators tend to align parallelly when they are bent in the same direction but vertically when they are not.

For the time scale during assembly, we measured the assembly time and the distance between two approaching actuators upon UV irradiation with different intensities (Supplementary Fig. 13). With the increase of the radiation intensity from 90 to 170 mW cm^{-2}, the time required for completing the aggregation decrease from approx. 9 s to approx. 4 s. The moving speed of the two approaching actuators is almost linear in early stage of the assembly, and increases exponentially during the later stage, which is consisted with the growing trend of the capillary attraction force during the assembling.

Supplementary Methods

Supplementary Methods 1

The solvents, dichloromethane, and deuterated chloroform, ($CDCl_3$) were purchased from Adamas and used as received. The photo initiator Bis(2,6-difluoro-3-(1-hydropyrro-1-yl)-phenyl)titanocene (I784) was purchased from Zhengzhou Alfa Chemical Co., Ltd.), China. Photoresponsive azobenzene monomer, (4,4'-bis[9-(acryloyloxy) undecyloxy]azobenzene, DA11AB6) and the liquid crystal monomer (1,4-bis[4-(9-acryloyloxynonyloxy)benzoyloxy]-2-methylbenzene, C9A) was purchased from Beijing Realchem Technology Co., Ltd. Materials were purchased from Fuhong Nano Materials Co., Ltd. (NM Microspheres, 30 μm), 3M (Electronic Fluid FC-70).

Supplementary Methods 2

DSC thermograms were obtained using TA Discovery DSC 250. The alignment of the LCPs was verified by using polarized optical microscopy (Leica DM2700P). The surface temperature of the actuator before and after UV irradiation was measured by an infrared thermometer (A665sc, FLIR).

Supplementary References

1. Yu, Y. L. & Ikeda, T. Alignment modulation of azobenzene-containing liquid crystal systems by photochemical reactions. *J. Photoch. Photobio. C.* **5**, 247-265 (2004).

2. Kondo, M., Yu, Y. L. & Ikeda, T. How Does the initial alignment of mesogens affect the photoinduced bending behavior of liquid-crystalline elastomers? *Angew. Chem. Int. Ed.* **45**, 1378-1382 (2006).

3. Yoshino, T. et al. Three-dimensional photomobility of crosslinked azobenzene liquid-crystalline polymer fibers. *Adv. Mater*. **22**, 1361-1363 (2010).

4. Dunn, M. L. Photomechanics of mono-and polydomain liquid crystal elastomer films. *J. Appl. Phys.* **102**, 013506 (2007).

5. Yun, J. H., Li, C., Chung, H., Choi, J., & Cho, M. Multiscale modeling and its validation of the trans-cis-trans reorientation-based photodeformation in azobenzene-doped liquid crystal polymer. *Int. J. Solids. Struct.* **128**, 36-49 (2017).

6. Ikeda, T., Nakano, M., Yu, Y., Tsutsumi, O., & Kanazawa, A. Anisotropic bending and unbending behavior of azobenzene liquid-crystalline gels by light exposure. *Adv. Mater.* 15, 201-205 (2003).

7. Vella, D. & Mahadevan, L. The “Cheerios effect”. *Am. J. Phys.* **73**, 817-825 (2005).

8. Yu, Y., Guo, M., Li, X. & Zheng, Q.-S. Meniscus-climbing behavior and its minimum free-energy mechanism. *Langmuir* **23**, 10546-10550 (2007).

9. Hu, D. L. & Bush, J. W. M. Meniscus-climbing insects. *Nature* **437**, 733-736 (2005).
