# Supplementary material for: Optocapillarity-driven assembly and reconfiguration of liquid crystal polymer actuators
Source: Nat Commun. 2020 Nov 13;11:5780. doi: 10.1038/s41467-020-19522-1 (PMC7666155; doi:10.1038/s41467-020-19522-1)
Supplement: Supplementary file 3 — Description of Additional Supplementary Files [file 41467_2020_19522_MOESM3_ESM.pdf]

Description of Additional Supplementary Files

Supplementary Movie 1: Optocapillarity-driven assembly and reconfiguration.

Description:

Part 1. Optocapillary-driven assembly of two shape-programmed actuators

Part 2. Optocapillary-driven reconfiguration of the assembly of two shape-programmed actuators

Supplementary Movie 2: Robustness of the assembled structure.

Description: Three actuators assembled into a linear structure after UV irradiation. Two of the three actuators were pulled away to break the linear structure by using two tweezers. After release of two separated actuators, the three actuators rapidly reassembled into the linear structure. The intensity of 365-nm and 470-nm light is 175 mW cm^{-2} and 150 mW cm^{-2}, respectively.

Supplementary Movie 3: Reversible assembly with well-defined selectivity.

Description:

Part 1. Optocapillary-driven reversible assembly of multiply actuators at air/water interface

Part 2. Selective aggregation controlled by light

Supplementary Movie 4: Optocapillarity-driven separation of a single actuator from its assembled structure.

Description: Four actuators were assembled to form an ordered structure upon the irradiation of 365-nm light. Using 470-nm light to illuminate one of the four actuators can make it to recover the flat state and separate away from the structure. The intensity of 365-nm and 470-nm light is 175 mW cm^{-2} and 150 mW cm^{-2}, respectively.

Supplementary Movie 5 Optocapillarity-driven switching of the chirality of assembled structures.

Description: The intensity of the ultraviolet laser and visible laser is 60 mW cm^{-2} and 45 mW cm^{-2}, respectively. The size of rectangular actuators is 6 mm × 2 mm × 0.03 mm.

Supplementary Movie 6: Meniscus climbing and rotating.

Description: When irradiated with UV light (175 mW cm^{-2}), a flat actuator placed at the foot of the meniscus bent downward and rotated to align itself perpendicular to the meniscus, and then accelerated and ascended the meniscus. And following irradiation of 470-nm light (150 mW cm^{-2}), the actuator unbent and slid down the meniscus.

Supplementary Movie 7: Versatility of optocapillary-driven assembly

Description:

Part 1. Optocapillary-driven reconfiguration of four rectangular actuators assembling into structures like the tetrominoes of the popular video game Tetris.

Part 2. Simple transformation

Part 3. Complex transformation

The transformation of the structures was achieved by alternative localized-irradiation of 450-nm and 360-nm laser with modulation of the incident direction. The intensity of the ultraviolet laser and visible laser is 60 mW cm^{-2} and 45 mW cm^{-2}, respectively.

Supplementary Movie 8: Optocapillarity-driven programmable reconfigurable

Description: Upon light irradiation, the actuators assembled into regular structures. The transformation between any two structures was achieved by alternative localized irradiation of 450-nm and 360-nm laser as well as modulation of the incident direction of the laser. The intensity of the ultraviolet laser and visible laser is 60 mW cm^{-2} and 45 mW cm^{-2}, respectively. The size of rectangular actuators is 6 mm $\times$ 2 mm $\times$ 0.03 mm.

Supplementary Movie 9: Optocapillarity-driven assembly of actuators at multiply fluid interfaces.

Description: Two triangular actuators and a rectangular actuator were placed at the air-water interface while three rectangular actuators were placed at the water/FC-70 interface below the air-water interface. Upon UV irradiation, the actuators assembled into programmed 3D ordered structures. The transformation of the structures was achieved by alternative localized irradiation of 450-nm and 360-nm laser with modulation of the incident direction. The intensity of the ultraviolet laser and visible laser is 60 mW cm^{-2} and 45 mW cm^{-2}, respectively.

Supplementary Movie 10: Optocapillarity-driven collaborative assemblies of the actuators at two adjacent fluid interfaces.

Description: Two rectangular actuators were placed at the air-water interface while a longer rectangular actuator and another two rectangular actuators was placed at the water/FC-70 interface below the air-water interface. Upon UV irradiation the five actuators assembled into a hierarchical structure. The intensity of the ultraviolet laser and visible laser is 60 mW cm^{-2} and 45 mW cm^{-2}, respectively.
